# 

**Published:** 1909-02

**Authors:** 


PUBLISHED MONTHLY. ATLANTA SUBSCRIPTION $1.00

### JOURNAL-RECORD OF MEDICINE

LIBRARY
Indexed
S. G. O.

Successor to { Atlanta Medical and Surgical Journal, Established 1855. and Southern Medical Record, Established 1870. } 53d Year

Vol. XI. ATLANTA, GA., FEBRUARY, 1909. No. 11

